# Hemoadsorption in Complex Cardiac Surgery—A Single Center Experience

**DOI:** 10.3390/jcm11237005

**Published:** 2022-11-27

**Authors:** Murali Manohar, Vivek Jawali, Siddu Neginahal, Sudarshan GT, Geetha Muniraj, Murali Chakravarthy

**Affiliations:** 1 Cardiac Surgery Department, Fortis Hospital, Bengaluru 560076, India; 2 Cardiac Sciences Board, Fortis Hospital, Bengaluru 560076, India; 3 Cardiac Surgery Perfusion Department, Fortis Hospital, Bengaluru 560076, India; 4 Anaesthesia Department, Fortis Hospital, Bengaluru 560076, India

**Keywords:** complex cardiac surgery, aortic surgery, redo, hyperinflammation, hemoadsorption, cytosorb, blood purification, cytokines

## Abstract

(1) Background: Cardiac surgery may evoke a generalized inflammatory response, typically magnified in complex, combined, redo, and emergency procedures with long aortic cross-clamp times. Various treatment options have been introduced to help regain control over post-cardiac surgery hyper-inflammation, including hemoadsorptive immunomodulation with CytoSorb^®^. (2) Methods: We conducted a single-center retrospective observational study of patients undergoing complex cardiac surgery. Patients intra-operatively treated with CytoSorb^®^ were compared to a control group. The primary outcome was the change in the vasoactive-inotropic score (VIS) from pre-operatively to post-operatively. (3) Results: A total of 52 patients were included in the analysis, where 23 were treated with CytoSorb^®^ (CS) and 29 without (controls). The mean VIS increase from pre-operative to post-operative values was significantly lower in the CS group compared to the control group (3.5 vs. 5.5, respectively, *p* = 0.05). In-hospital mortality in the control group was 20.7% (6 patients) and 9.1% (2 patients) in the CS group (*p* = 0.26). Lactate level changes were comparable, and the median intensive care unit and hospital lengths of stay were similar between groups. (4) Conclusions: Despite notable imbalances between the groups, the signals revealed point toward better hemodynamic stability with CytoSorb^®^ hemoadsorption in complex cardiac surgery and a trend of lower mortality.

## 1. Introduction

Cardiac surgery may evoke a generalized inflammatory response in patients, with sometimes serious clinical consequences, despite advances in pharmacology, perfusion technology, cardiovascular monitoring, and anesthetic and surgical techniques [1]. As a result of an exponential increase in percutaneous coronary interventions (PCI) and transcatheter aortic valve implantations in recent years, many more patients with a higher surgical risk are undergoing cardiac surgery than ever before [2,3]. Furthermore, complex, combined, and emergency procedures, long aortic cross-clamp times, and re-operations increase the risk of complications and mortality [4].

Even with significant technical improvements in recent years, cardio-pulmonary bypass (CPB) itself remains a frequent trigger for the development of a dysregulated hyper-inflammatory host response, similar to sepsis. This is accompanied by elevated levels of inflammatory mediators and may result in a compromised microcirculation, increase in lactate levels, vasoplegia and subsequent organ dysfunction [5]. Persistent hyperlactatemia, together with high doses of vasoactive medications in the post-operative period are found to be associated with higher mortality [4].

Various techniques, technological advancements, and treatment options have been introduced during recent decades with the aim of helping to regain control over this post-cardiac surgery hyper-inflammation (in the literature, this is often described as systemic inflammatory response syndrome or SIRS). The hemoadsorption of inflammatory mediators has recently emerged as a promising strategy with the goal to attenuate the deleterious effects of the dysregulated inflammatory response while preserving the ability of the patient’s immune system to mount an appropriate defense against these challenges during the peri-operative period [6].

One of the most widely investigated immunomodulating hemoadsorptive treatments is CytoSorb^®^. The aim of our study is to evaluate CytoSorb^®^ therapy in the setting of complex cardiac surgery.

## 2. Materials and Methods

The present study considers a single-center, retrospective observational study of patients undergoing complex cardiac surgery. Patients with intra-operative use of the hemoadsorption device CytoSorb^®^ (CS group) were compared to a control group without intra-operative CytoSorb^®^ use (Control group). Inclusion criteria were: (1) patients with a CPB time longer than 120 min and (2) the procedure was labeled as “complex” by the operating team, or was a redo procedure. The observational period was between December 2018 and October 2021. Informed consent was waived due to the retrospective nature of this study.

CytoSorb^®^ therapy (CytoSorb^®^ 300 mL Device, CytoSorbents Inc., Princeton, NJ, USA) is a blood purification technique based on the adsorption of hydrophobic molecules of up to approximately 60 kDa of molecular weight. It is installed in an extracorporeal circuit of various platforms and used for the removal of excess levels of cytokines, together with some metabolites such as bilirubin and myoglobin, and also antithrombotic medications, such as ticagrelor and/or rivaroxaban [7]. As the adsorption process is concentration-dependent [8], the removal of clinically relevant levels of target molecules primarily occurs with excessively elevated plasma concentrations of these molecules.

The primary outcome of the present study was the difference in the vasoactive-inotropic Score (VIS) change pre-operatively to post-operatively. The post-operative calculation of VIS comprised of the highest levels of respective vasoactive medications that patients received within the first 24 h after the end of surgery. Secondary outcomes included changes in lactate levels, nosocomial infections, bleeding and blood product consumption, mechanical ventilation duration, continuous renal replacement therapy (CRRT) requirement, intensive care unit (ICU) and hospital length of stay, and in-hospital mortality.

Data were analyzed using SPSS software version 25 (SPSS Inc., Chicago, IL, USA). After testing the data for normality using the Shapiro–Wilk test, *t*-tests (CI 95%) for normally distributed variables and Wilcoxon rank-sum tests (IQR 25–75 percentiles) for non-normally distributed variables were used. The threshold for statistical significance was a *p*-value of 0.05 with a value <0.05 considered significant. Categorical data were expressed as the number of patients and frequencies, and were compared using the chi-square test.

## 3. Results

A total of 52 patients who underwent open-heart surgery on CPB were included in the analysis. Almost all patients required combined procedures comprising multiple valve surgery, coronary artery bypass grafting (CABG), and various procedures involving the aorta. Twenty-three patients were treated with CytoSorb^®^ (CS) and 29 without (controls). Overall, the patients in the control group were younger when compared to the CS group, where the median age was 57 years [47–61] vs. 64 years [60–68] in the CS group (*p* = 0.01). The patients were predominantly male in both groups (61% in the CS group and 66% in the control group). For almost half of the control group (45%), this was a redo procedure, while only 22% of the CS group included re-operations (*p* = 0.08). The median cross-clamp time was 121.5 min [100–156] in the CS group but was significantly longer in the control group, 154 min [131.5, 195.5] (*p* = 0.04). Another significant difference was in the pre-operative usage of an intra-aortic balloon pump (IABP) which was present in almost 22% of patients in the CS group while no patients among the controls needed this treatment before the operation (21.7 % vs. 0%, respectively, *p* < 0.01). The baseline characteristics of all patients are given in Table 1.

**Figure 1:**
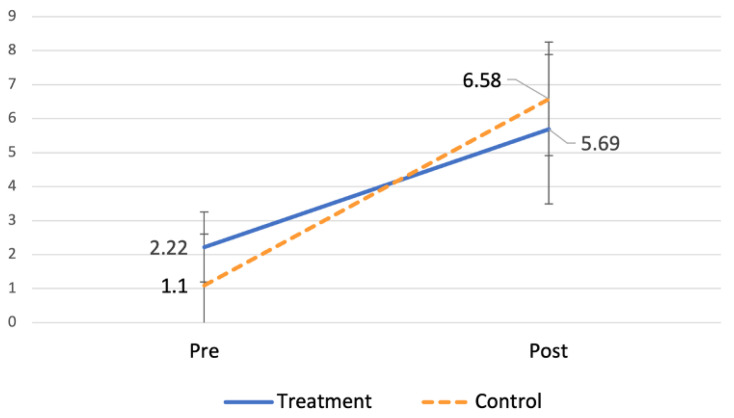
VIS change pre- versus post-operation.

The vasoactive-inotropic score increased post-operatively in both groups (Figure 1).

**Figure 2:**
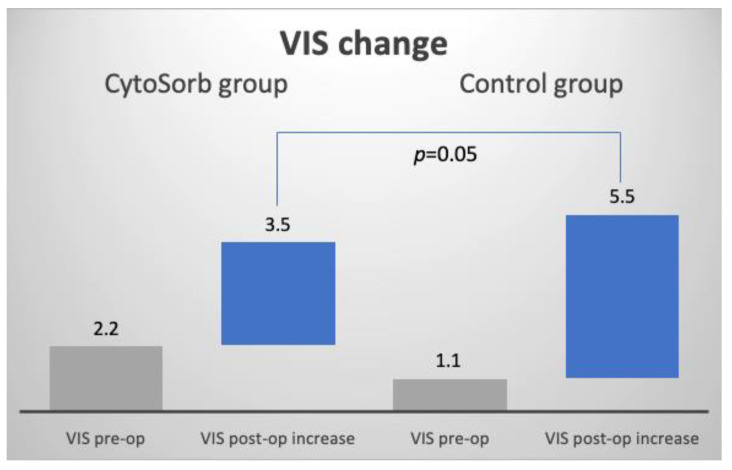
Comparison of difference in VIS change: CytoSorb^®^ + 3.5 vs. control + 5.5, *p* = 0.05.

The mean VIS increase (Figure 2) from pre-op to post-op was significantly lower in the CS group compared to the control group, with 3.5 vs. 5.5, respectively, *p* = 0.05 (Table 2).

In-hospital mortality in the control group was 20.7% (6 patients) and 9.1% (2 patients) in the CS group, however, this difference did not reach statistical significance (*p* = 0.26), as did not the difference in the post-operative VIS - 5.7 with CytoSorb^®^ vs. 6.6 without hemoadsorption (*p* = 0.53). The median ICU and hospital lengths of stay were similar between groups. Lactate levels for both groups increased after 6 h post-operatively, however, they decreased comparably over the following 24 hrs. Both groups were similar in terms of the mechanical ventilation time and CRRT requirement (Table 3). Two patients in the control group had a nosocomial infection *versus* no patients in the CytoSorb^®^ group. One patient needed re-exploration due to bleeding in the CytoSorb^®^ group.

There were no device-related adverse events observed. 

## 4. Discussion

In recent years, the morbidity of cardiac surgical patients has increased, mainly due to increased utilization of cardiac surgery in elderly and more vulnerable patients with increasing rates of pre-existing diseases. This has been found to be associated with post-operative organ dysfunction and prolonged mechanical ventilation, leading to more complex and prolonged intensive care treatment, and thus impaired outcomes [9]. It has been shown that patients who undergo cardiac surgery who have experienced multiple complications post-operatively have substantial differences in their pre-operative characteristics (i.e., preexisting comorbidities and older age) when compared with those who recovered without complications or with only an isolated complication [10]. Furthermore, the need for re-operation has been found to be an independent predictor of both short-term and long-term mortality after a redo cardiac surgery, especially in the setting of open-heart surgery with a re-sternotomy and the utilization of CPB [11,12].

In complex cardiac operations involving combined surgical procedures and redo operations with prolonged CPB times, a dysregulated systemic immune response may occur. This systemic inflammatory response is orchestrated in a large part by high levels of circulating cytokines, a phenomenon termed as a “cytokine storm” in more severe cases [13]. Such severe hyper-inflammation is often associated with a higher incidence of renal failure, hemodynamic instability, bleeding complications, and thrombotic events, which all together lead to higher mortality in these patients [14,15].

Vasoplegia is a frequent complication following cardiac surgery, especially in the post-operative course. It is a syndrome defined as abnormal/low systemic vascular resistance (SVR) manifesting as severe hypotension, with normal or elevated cardiac index (CI). Some patients develop vasoplegic shock, involving deleterious vasodilation and hypotension, even with normal CI and adequate fluid resuscitation, leading to reduced tissue perfusion and metabolic acidosis. First-line treatment includes high-dose vasopressors, particularly catecholamines, to maintain adequate mean arterial pressure. Vasoplegic shock that is refractory to these agents is detrimental [16]. Rescue therapy is urgently needed in refractory cases to limit prolonged hypoperfusion, maintain adequate organ function, and reduce morbidity and mortality [5,16].

Considering the substantial proportion of frail patients with multiple comorbidities in cardiac surgery today, the ever-present CPB-induced systemic hyper-inflammation, together with the high rate of complex and redo procedures, we hypothesized that intra-operative CytoSorb^®^ therapy might significantly reduce hyper-inflammation and improve hemodynamic stability in patients undergoing complex cardiac surgery.

CytoSorb^®^ therapy is not supposed to suppress the physiological inflammatory response, but rather aid the immune system to regain control and modulate its dysregulated response. Furthermore, while removing overshooting levels of cytokines, the concurrent removal of trigger molecules, such as PAMPs (pathogen-associated molecular patterns) and DAMPs (damage-associated molecular patterns), which reside in the above-described adsorption range, have been observed [17]. In this way, CytoSorb^®^ therapy aims at helping the patient’s body mitigate the cytokine hyper-release cytotoxic effects and attenuate the dysregulated inflammatory response, intending to prevent the progression of organ dysfunction.

One of the most prominently reported outcomes in clinical investigations with CytoSorb^®^ is the reduction in vasopressor demand [18,19,20,21,22,23,24]. Therefore, we decided to use the vasoactive-inotropic score (VIS) change pre-operatively to post-operatively as our primary endpoint. VIS has been validated in adult cardiac surgery and has been found to be an independent predictor of combined morbidity and mortality [25,26] with apparently promising prospects in future study designs [27]. Considering the effect of a decreased need for vasoactive medications concomitant with the CytoSorb^®^-mediated attenuation of hyper-inflammation associated vasoplegia in our patients, we expected the VIS change from pre-op to post-op to be improved in the CytoSorb^®^ group compared to controls. The results of our study show that the post-operative VIS increase was, in fact, significantly lower in the CytoSorb^®^ group, indirectly confirming that hemoadsorption of excessive levels of cytokines prevented an overwhelming development of vasoplegia, potentially also influencing mortality. Survival was numerically better in the CS group than in the control group (91% vs. 79%, respectively), but this difference was not statistically significant within this small cohort. The present study was designed as a pilot study at our center when we started with this innovative adjunctive therapy in 2018.

In two very recent single-center controlled studies involving high-risk infective endocarditis (IE) patients, the authors could show that intra-operative hemoadsorption lowered the incidence of sepsis, sepsis-related death [28], and overall in-hospital mortality, with a better post-operative clinical course [29]. Two studies on aortic surgery patients in which CytoSorb^®^ was used intra-operatively demonstrated significantly better hemodynamic stability and shorter lengths of ICU and hospital stays [19], as well as decreased usage of norepinephrine and blood products compared to controls [20]. It has been reported that transplanted patients may also benefit from intra-operative hemoadsorption therapy, reducing their need for vasopressors, organ support, and the rate of primary graft failure [21]. To our knowledge, there are only two case series in the cardiac surgery setting that have reported on VIS before and after CytoSorb^®^ treatment, but hemoadsorption was delivered post-operatively, utilizing either continuous renal replacement therapy (CRRT) or extracorporeal membrane oxygenation (ECMO) as the delivery platform. In both studies, VIS significantly decreased after CytoSorb^®^ therapy was administered in patients who developed septic shock post cardiac surgery [22] or in other critically ill patients [30]. Our study is the first to report on a difference in VIS in complex cardiac surgery patients, comparing those treated intra-operatively with hemoadsorption on CPB to those without.

None of the aforementioned secondary outcomes were different between the two groups in our study. The plausible reasons for this most probably lie in the fact that the two groups were not well balanced. Mortality, for example, was substantially higher in controls, but it has to be taken into account that the aortic cross-clamp time was significantly longer and re-sternotomies were twice as frequent in this group. On the other hand, the need for IABP was exclusively present in the CS group reflecting the high acuity of such patients. Moreover, the EuroSCORE II was notably higher in the treatment group, the pre-operative VIS was twice as high as in control patients, and the CytoSorb^®^ patients were significantly older. Nevertheless, the results of this study represent real-world data, being a report on a single-center experience, the first of this kind in India, that, in our opinion, can serve as a valuable guideline for designing future large prospective trials investigating the role of hemoadsorption in complex cardiothoracic surgery.

Another important indication which was not covered in this analysis is the increasing use of antithrombotic medications, especially in high-risk and elderly patients. A noteworthy proportion of such patients suffer from non-valvular atrial fibrillation and might undergo a simultaneous maze procedure or occlusion of the left atrial appendage. As these patients are commonly treated with DOACs (direct-acting oral anticoagulants) or P2Y_12_ inhibitors, such as ticagrelor (after PCI), intra-operative hemoadsorption holds promise to prevent bleeding complications [31,32].

There were several significant baseline differences between the groups causing a notable imbalance which represents a serious limitation of our study. The lack of sequential VIS values beyond the first post-operative day may be considered as another shortcoming, however, we believe that the potential effects of intra-operative hemoadsorption would have a rather short term effect that would be seen immediately after the surgery and that the further course in the ICU may be influenced by many other factors which would introduce even more confounders. Therefore, the post-operative VIS calculation was limited to the first 24 h only. Moreover, the retrospective nature and small sample size, together with possible patient selection bias all call for caution when interpreting the results of this study.

Notwithstanding the above, this is the largest case-control study evaluating complex cardiac surgery patients treated with intra-operative hemoadsorption in India so far and the signals revealed with the performed analysis may guide future investigations in this clinical setting.

## 5. Conclusions

To our knowledge, this is the largest case-control study in the setting of complex cardiac surgery involving various, mostly combined procedures, that has evaluated the effects of cytokine hemoadsorption in a high-risk patient population in India. As such, it represents real-world data, albeit with several important limitations (already discussed). However, it also provides excellent insight into the potential of CytoSorb^®^ to improve clinical outcomes and is a guide for the design of future studies. In the present study, signals point toward better hemodynamic stability with the use of CytoSorb^®^ in complex cardiac surgery, and a trend towards lower mortality. However, the role of hemoadsorption and the potential routine implementation of this approach requires further evaluation in prospective studies.

## Figures and Tables

**Table 1:** Baseline characteristics.

Mean (CI) or Median [IQR]				
Variables	Overall (n = 52)	CS Group (n = 23)	Control Group (n = 29)	*p*-Value
Age, years	61 [51.5, 65.5]	64 [60.0, 68.0]	57 [47.0, 61.0]	0.01
Sex–male, % (n)	63.5 (33)	60.9 (14)	65.5 (19)	0.73
Redo procedures, % (n)	34.6 (18)	21.7 (5)	44.8 (13)	0.08
Lactate mmol/L	1.36 [1.0, 2.1]	1.25 [0.8, 2.1]	1.40 [1.1, 2.1]	0.38
EuroSCORE II, %	4.6 [2.2, 14.2]	7.3 [2.7, 21.4]	4.4 [1.5, 9.2]	0.17
Mechanical ventilation, % (n)	15.4 (8)	21.7 (5)	10.3 (3)	0.26
Intra-aortic balloon pump (IABP), % (n)	9.6 (5)	21.7 (5)	0	<0.01
Continuous renal replacement therapy (CRRT), % (n)	1.9 (1)	4.3 (1)	0	0.26
Inotropes, % (n)	25.0 (13)	34.8 (8)	17.2 (5)	0.15
Hemoglobin (Hb), g/dL	12.0 [10.2, 13.5]	11.5 [9.5, 12.8]	12.6 [11.2, 13.8]	0.08
White blood cell count (WBC), N × 10^9^/L	9.2 (6.3, 11.9)	8.2 (5.9, 11.4)	10.6 (6.4, 12.0)	0.64
Vasoactive-inotropic score (VIS)	1.6 (0.7, 2.5)	2.2 (0.7, 3.7)	1.1 (0.1, 2.1)	0.20
CPB time, min	211 [167.5, 275.5]	217 [171.0, 264.0]	211 [164.0, 278.0]	0.99
Cross-clamp time, min	143 [115.0, 181.0]	122 [100.0, 156.0]	154 [131.5, 195.5]	0.04
CPB temperature, °C	31 [28, 32]	30 [28, 32]	32 [28, 32]	0.30

Data are presented as mean (CI – confidence interval), median [IQR—interquartile range], or proportion expressed in % and number (n). Statistically significant *p*-values are in bold.

**Table 2:** Vasoactive-inotropic score change.

Mean (CI Difference)				
Variable	CS Group	Control Group	Delta	*p*-Value
VIS mean change ^1^	3.5 (2.2, 4.8)	5.5 (3.6, 7.4)	2 (−0.4, 4.4)	0.05

^1^ Vasoactive-inotropic score mean change: the difference in post-op and pre-op mean.

**Table 3:** Secondary outcomes.

Median [IQR], Mean (CI), or Proportion % (n)				
Variable	Overall	CS Group	Control Group	*p*-Value
Mortality, % (n)	15.7 (8) *	9.1 (2) *	20.7 (6)	0.26
VIS post-op	6.2 (4.8, 7.5)	5.7 (4.2, 7.2)	6.6 (4.5, 8.7)	0.53
LoS, days ICU Ward ICU + Ward				
	3 [2, 5]	4 [3, 6]	3 [2, 4]	0.28
	2.5 [1, 4]	3 [2, 4]	2 [1, 3]	0.14
	6 [5, 8]	6 [5, 8]	6 [5, 8]	0.74
CRRT post-op, % (n)	9.6 (5)	8.7 (2)	10.3 (3)	0.84
Mechanical ventilation post-op, % (n)	96.2 (50)	95.7 (22)	96.6 (28)	0.87
Duration of mechanical ventilation, days	1 [1, 2]	1 [1, 2]	1 [1, 2]	0.83
Lactates post-op on admission 6 h 12 h 24 h				
	2.3 [1.6, 3.1]	1.8 [1.3, 3.1]	2.7 [−3.1, 1.1]	0.13
	3.5 [2.0, 6.0]	3.8 [2.5, 7.8]	3.2 [1.9, 5.8]	0.24
	2.3 [1.4, 4.6]	2.9 [1.9, 4.6]	1.8 [1.4, 3.0]	0.15
	1.8 [1.3, 3.7]	1.9 [1.4, 3.2]	1.6 [1.1, 3.8]	0.52
Total blood loss, mL intra-op post-op combined				
	759 [591, 1181]	903 [647, 1200]	715 [445, 1089]	0.08
	570 [400, 920]	700 [460, 990]	510 [400, 640]	0.12
	1512 [1046, 2019]	1475 [998, 1941]	1677 [1115, 2337]	0.23
Blood product usage, units pRBC FFP Platelets Cryoprecipitate				
	4 [3, 5]	4 [3, 6]	4 [2, 5]	0.26
	0 [0, 4]	0 [0, 3]	1 [0, 4]	0.38
	0 [0, 0]	0 [0, 0]	0 [0, 4]	0.48
	0 [0, 0]	0 [0, 0]	0 [0, 0]	0.28

Data are presented as mean (CI – confidence interval), median [IQR- interquartile range], or proportion expressed in % and number (n). VIS: vasoactive-inotropic score, LoS: length of stay, CRRT: continuous renal replacement therapy, pRBC: packed red blood cells, FFP: fresh frozen plasma. * Missing data for in-hospital mortality status of one patient.
